# A retrospective cohort study comparing the effects on inflammatory response and clinical outcomes of vancomycin-PMMA versus vancomycin-sulfate calcium in chronic osteomyelitis

**DOI:** 10.1080/07853890.2026.2680392

**Published:** 2026-06-09

**Authors:** Shuman Han, Xirui Wu, Tianhao Wu

**Affiliations:** a Department of Radiology, Hebei Medical University Third Hospital, Shijiazhuang, Hebei, China; b Department of Orthopedics, Hebei Medical University Third Hospital, Shijiazhuang, Hebei, China

**Keywords:** Chronic osteomyelitis, vancomycin, polymethylmethacrylate, calcium sulfate, infection control, inflammatory

## Abstract

**Purpose:**

This study aimed to compare the impact on inflammatory response, clinical efficacy, and safety between vancomycin-loaded polymethylmethacrylate (PMMA) and a combination of vancomycin-loaded PMMA with vancomycin sulfate calcium (PMMA-SC) in treating chronic osteomyelitis.

**Methods:**

A retrospective cohort study involved 244 patients with chronic osteomyelitis treated between January 2017 and December 2024. Patients were divided into PMMA (*n* = 126) and PMMA-SC (*n* = 118) groups. Postoperative outcomes, efficacy, inflammatory markers, quality of life, and complications were evaluated.

**Results:**

The PMMA-SC group demonstrated significantly higher rates of infection control (87.29% vs. 72.22%, *p* = 0.004) and lower reoperation rates (7.63% vs. 21.43%, *p* = 0.002) compared to the PMMA group. The PMMA-SC intervention was associated with a more robust attenuation of systemic inflammation, evidenced by significantly greater reductions in neutrophil-to-lymphocyte ratio (NLR), platelet-to-lymphocyte ratio (PLR), systemic immune-inflammation index (SII), erythrocyte sedimentation rate (ESR), C-reactive protein (CRP), and procalcitonin (PCT) (all *p* < 0.05). Quality of life measures, particularly physical functioning, general health, and vitality, also favored the PMMA-SC group (*p* < 0.05). The overall complication rate was significantly lower in the PMMA-SC group (8.47% vs. 26.19%, *p* < 0.001). The multivariate logistic regression analysis demonstrated that PMMA-SC was a significant protective factor for treatment success (*p* < 0.05).

**Conclusion:**

The combination of vancomycin sulfate calcium with PMMA was associated with improved outcomes compared to PMMA alone, potentially attributable to its association with enhanced control of infection and a more favorable modulation of the systemic inflammatory and immune response. These findings support its consideration as a promising therapeutic strategy for chronic osteomyelitis.

## 1. Introduction

Chronic osteomyelitis, a persistent and challenging infection of the bone and surrounding tissue, represents a significant clinical conundrum, often resulting from an acute osteomyelitis episode that fails to resolve completely [1,2]. It is characterized by recurrent exacerbations, sequestrum formation, and the presence of bacterial biofilms, which not only render systemic antibiotic therapies less effective but also perpetuate a state of dysregulated and persistent local inflammation [3]. The management of chronic osteomyelitis necessitates a multifaceted approach, incorporating surgical intervention to remove necrotic tissue and the administration of potent antimicrobials delivered in a way that penetrates the site of infection effectively [4,5].

The rising prevalence of methicillin-resistant *Staphylococcus aureus* (MRSA) alongside other resistant organisms has propelled vancomycin to the forefront of osteomyelitis treatment regimens [6]. Vancomycin, a glycopeptide antibiotic, was particularly efficacious against Gram-positive pathogens, which were commonly implicated in osteomyelitic infections [7–9]. However, the systemic administration of vancomycin was often hindered by issues of poor bone penetration, systemic toxicity, and the need for prolonged therapy durations. Local antibiotic delivery systems have thus been developed to address these limitations, providing targeted, high-concentration antimicrobial therapy directly to the site of infection while minimizing systemic exposure. The efficacy of such local therapies is evaluated not only by clinical outcomes but also by their ability to resolve the systemic inflammatory burden [10].

Polymethylmethacrylate (PMMA) and calcium sulfate were two local delivery systems that have gained prominence for their utility in osteomyelitis management [11]. PMMA, a biostable polymer, acts as a carrier for vancomycin, allowing for sustained-release antibiotic therapy. It’s been used extensively due to its robust mechanical properties, which can also offer structural support in void-filling applications post-debridement [12]. However, one major drawback of PMMA was its non-biodegradable nature, necessitating surgical removal after the antibiotic has been eluted, potentially increasing patient morbidity and healthcare costs [13].

Conversely, calcium sulfate was a fully biodegradable material well-tolerated by the human body, alleviating the need for subsequent removal surgeries [14]. It serves as both an antibiotic carrier and an osteoconductive scaffold, facilitating new bone growth as it dissolves. The rapid elution of vancomycin from calcium sulfate can achieve high initial local antibiotic concentrations, while its complete bioresorption may reduce foreign-body reactions and promote osteoconduction. When combined with PMMA, which provides sustained antibiotic release and structural void filling, the two carriers may exert complementary effects on infection clearance and modulation of the inflammatory milieu [15,16]. Despite its apparent advantages, the relatively rapid dissolution rate of calcium sulfate compared to PMMA can lead to concerns regarding the sustained delivery and adequate dosing of vancomycin over the requisite treatment periods. Given their distinct physicochemical properties, it is plausible that these two carrier systems may differentially influence the local and systemic inflammatory milieu post-treatment. However, head-to-head comparisons of these two carrier systems regarding their impact on systemic inflammatory response and clinical outcomes in chronic osteomyelitis remain limited.

This study seeks to assay the efficacy and safety profiles of vancomycin delivered *via* PMMA compared to calcium sulfate, with a particular focus on their comparative impact on the response of systemic inflammation and clinical outcomes in treating patients with chronic osteomyelitis. We recognize that this study involves a historical comparison, as the two treatment strategies were introduced in different time periods.

## 2. Materials and methods

### 2.1. Selection criteria

The study included patients who met the diagnostic criteria for chronic post-traumatic or postoperative osteomyelitis [17], were 18 years or older, had bacterial cultures sensitive to vancomycin, completed a minimum of one year of follow-up, and possessed comprehensive medical records and follow-up data.

Chronic post-traumatic or postoperative osteomyelitis was defined as bone infection persisting for more than six weeks following an open fracture or orthopedic surgical procedure, confirmed by clinical signs (persistent pain, sinus tract drainage), radiological evidence (sequestrum formation, cortical destruction), and positive microbiological culture or histopathological findings from intraoperative bone specimens. This definition aligns with the established consensus criteria for fracture-related infection (FRI), which are widely recognized in the literature.

Exclusion criteria were applied to patients with hematogenous osteomyelitis or acute post-traumatic or postoperative osteomyelitis; those with concurrent orthopedic conditions such as diaphyseal osteosarcoma, histiocytosis, septic arthritis, or osteoid osteoma; and individuals with serious medical or surgical diseases, tuberculosis, tumors, Parkinson’s disease, or rheumatoid arthritis. Additional exclusions included patients with surgical contraindications or signs of malignant lesions.

This study obtained approval from the Institutional Review Board and Ethics Committee of our institution. As the research solely involved de-identified patient data, minimizing any risk or impact on the patients, the requirement for informed consent was waived. This waiver was approved by the Institutional Review Board and Ethics Committee in compliance with regulatory and ethical standards for conducting retrospective research.

### 2.2. Study population and group allocation

A retrospective analysis was conducted on 244 patients diagnosed with chronic osteomyelitis who were admitted to our hospital between January 2017 and December 2024. A total of 328 patients with suspected chronic osteomyelitis were initially screened. Of these, 84 were excluded due to lack of vancomycin-sensitive bacterial cultures (*n* = 51), incomplete follow-up data (*n* = 22), or other exclusion criteria (*n* = 11). The patients were categorized into two groups based on their treatment methods. The first group, consisting of 126 patients, was treated with vancomycin-loaded PMMA and designated as the PMMA group. The second group, comprised of 118 patients, received a combined treatment of vancomycin-loaded PMMA and vancomycin sulfate calcium, and was designated as the PMMA-sulfate calcium (PMMA-SC) group.

The choice between PMMA and PMMA-SC was based on surgeon preference and the period of treatment; PMMA was predominantly used between 2017–2020, while PMMA-SC was increasingly adopted from 2021 onwards. Additionally, larger bone defects or those requiring enhanced osteoconductive support tended to receive PMMA-SC at the surgeon’s discretion.

### 2.3. Treatment approach

All patients received surgical treatment for infection, which included the removal of internal fixation materials, extensive debridement of infected soft tissue and necrotic bone, resection of sclerotic bone within the infected area using a high-speed burr, cleaning with a wound pulse irrigation system, elimination of dead space, insertion of a drainage tube, and wound closure with subsequent refixation. In the PMMA group, the dead space was filled with a PMMA spacer mixed with vancomycin. For the PMMA-SC group, the dead space was managed using both a PMMA spacer mixed with vancomycin and calcium sulfate pellets. The basic surgical principles and sequence were consistent for all patients, except for the materials used to fill the bone space. Vancomycin was mixed at a standardized dose of 2 g per 40 g PMMA powder, and for calcium sulfate, 1 g vancomycin per 10 cc of granules was used. These dosages were consistent across all patients in both groups.

Closed suction drainage was placed in all patients for 24–48 h postoperatively. Although drainage theoretically reduces local antibiotic concentration, this practice was maintained for several reasons: (1) prevention of hematoma formation, which can serve as a bacterial culture medium and increase wound tension; (2) reduction of persistent wound drainage risk; and (3) evidence that the majority of antibiotic elution from both PMMA and calcium sulfate occurs within the first 48–72 h, with therapeutic local concentrations maintained despite drainage during this period. Drain removal was performed once output decreased to <50 mL per day.

In the PMMA group, after the removal of internal fixation materials and thorough debridement, a vancomycin-loaded PMMA spacer was placed into the bone defect prior to hardening. For patients in the PMMA group, the vancomycin-loaded spacer was removed in a planned second-stage surgery approximately 6–8 weeks after implantation, following completion of the local antibiotic elution period. This two-stage approach is standard practice in our institution to prevent bacterial colonization on the permanent non-biodegradable material.

For the PMMA-SC group, following removal of internal fixation materials and comprehensive debridement, the bone defect was managed using a specific layered technique: First, a single layer of vancomycin-loaded calcium sulfate granules (containing 1 g vancomycin per 10 cc of calcium sulfate) was placed directly onto the debrided bone surface to maximize osteoconductive contact. Second, vancomycin-loaded PMMA (mixed with 2 g vancomycin per 40 g PMMA powder) was prepared and, while still in a doughy state, carefully molded to fill the central portion of the cavity, ensuring it did not completely encase the calcium sulfate layer. Third, additional vancomycin-loaded calcium sulfate granules were placed on the superficial surface of the PMMA spacer, opposite the bone interface, without being embedded into the PMMA. This layered configuration allows the calcium sulfate to provide both deep osteoconductive support and superficial antibiotic elution, while the PMMA maintains structural void filling. The PMMA-SC combination was applied as part of a single-stage debridement and implantation procedure. However, because the PMMA component is non-biodegradable, patients in the PMMA-SC group also underwent planned second-stage surgery for PMMA removal at 6–8 weeks post-implantation. The calcium sulfate component was left *in situ* to undergo complete bioresorption, providing sustained antibiotic elution and osteoconductive support during the intervening period.

### 2.4. Data acquisition and efficacy evaluation

Patient data were collected from the medical record system and included demographic characteristics, baseline disease information, Cierny–Mader classification, postoperative outcomes, treatment efficacy, inflammatory markers, the Short Form-36 Health Survey Questionnaire (SF-36) scores, and complications.

The Cierny–Mader classification system [18] was used to categorize patients with chronic post-traumatic osteomyelitis: type 1 indicates intramedullary osteomyelitis, type 2 denotes superficial osteomyelitis, type 3 was classified as localized osteomyelitis, and type 4 represents diffuse osteomyelitis.

Infection controlled was defined as absence of clinical signs of infection (no local swelling, redness, warmth, sinus discharge, or fever) with normalization of inflammatory markers (CRP <5 mg/L, ESR <20 mm/h) at the two-month follow-up without additional antibiotic therapy. Persistent or recurrent infection was defined as failure to meet these criteria or reappearance of infection signs after initial control within the follow-up period.

Efficacy in managing chronic osteomyelitis was assessed in accordance with the 2008 guidelines from ‘Huang Jiasi Surgery’ [19], which classify outcomes into ‘healed,’ ‘improved,’ or ‘unhealed.’ A ‘healed’ status was defined by complete symptom resolution, normalized inflammatory markers, restored limb function, fully closed sinus tracts and wounds, uniform bone density on X-rays, absence of sequestrum or cavities, and no recurrence within one year. ‘Improved’ refers to reduced symptoms, partial normalization of inflammatory markers, partial recovery of limb function, satisfactory healing of sinus tracts and wounds, partial bone repair on X-rays, no residual sequestrum, and no recurrence within one year. The ‘improved’ category, while indicating significant clinical improvement, may in some cases represent a state where residual low-grade infection cannot be completely excluded; such patients require close long-term follow-up. ‘Unhealed’ status was characterized by persistent or worsening symptoms, abnormal inflammatory markers, ongoing instability or progression of lesions on X-rays, and presence of sequestrum or cavities. The total effective rate was calculated as the proportion of patients classified as either healed or improved. Outcome adjudication (healed/improved/unhealed) was performed by two independent orthopedic surgeons who were blinded to the treatment assignment, based on the predefined criteria.

### 2.5. Detection of inflammatory indicators

Fasting venous blood samples were collected from patients upon admission and two months post-treatment. For complete blood count analysis, 2 mL of venous blood was collected into EDTA-K2 anticoagulant tubes and analyzed using an automated hematology analyzer (XN-9000, Sysmex Corporation, Japan) to determine the counts of white blood cells, neutrophils, lymphocytes, and platelets. The neutrophil-to-lymphocyte ratio (NLR), platelet-to-lymphocyte ratio (PLR), and systemic immune-inflammation index (SII) were derived from these primary parameters. Simultaneously, a 2 mL blood sample was collected in a sodium citrate tube for erythrocyte sedimentation rate (ESR) measurement using an automated ESR analyzer (BS-2000M, URIT Medical Electronic Co., Ltd., China). Additionally, a 2 mL sample was collected in a serum separation tube, centrifuged at 3,000 rpm for 10 min, and the resultant serum was used to quantify C-reactive protein (CRP) and procalcitonin (PCT) levels *via* an automated electrochemical luminescence immunoassay system (Cobas e 801, Roche Diagnostics, Switzerland).

### 2.6. Assessment of quality of life (QoL)

The QoL of patients was evaluated one year after treatment using the Short Form-36 (SF-36) Health Survey. The questionnaire was completed by patients either during outpatient follow-up visits or *via* telephone interview conducted by trained research assistants. The SF-36 assesses eight dimensions: physical functioning (PF), role limitations due to physical health (RP), bodily pain (BP), general health perceptions (GH), vitality (VT), social functioning (SF), role limitations due to emotional problems (RE), and mental health (MH). Each dimension was scored on a scale from 0 to 100, with higher scores indicating better QoL. Cronbach’s alpha coefficients for all dimensions exceeded 0.70, demonstrating good internal consistency [20].

### 2.7. Statistical analysis

Data analysis was performed using SPSS version 29.0 (SPSS Inc., Chicago, IL, USA). Categorical variables were presented as frequencies and percentages [*n* (%)], chi-square tests and Fisher’s exact test were employed. Continuous variables were subjected to normality testing using the Shapiro–Wilk method. Variables following a normal distribution were expressed as mean ± standard deviation (M ± SD). The multivariate model assessed the independent association of treatment approach, age, diabetes and smoking history with the achievement of treatment effectiveness (combined ‘healed’ and ‘improved’ outcomes), after adjusting for all other variables.

To account for multiple comparisons, we considered a two-tailed *p* < 0.05 as statistically significant, and the results should be interpreted with caution due to potential type I error. The covariates of multivariate logistic regression model were selected based on their clinical relevance and potential confounding effects on treatment outcomes; the limited number of covariates was chosen to avoid overfitting given the sample size.

## 3. Results

### 3.1. Demographic characteristics

The comparison of demographic characteristics between the PMMA group and the PMMA-SC group (Table 1) revealed no significant differences across all parameters assessed. There were no significant differences in gender distribution, BMI, hypertension prevalence, diabetes prevalence, smoking history, drinking history, marital status, or educational level (*p* > 0.05) between the two groups. These findings indicate that the two treatment groups had similar demographic characteristics. However, as they represented consecutive cohorts from different time periods rather than a matched design, this similarity does not eliminate the risk of selection bias.

**Table 1.** Comparison of demographic characteristics between two groups.

Parameters	PMMA group (*n* = 126)	PMMA-SC group (*n* = 118)	*t*/*χ*^2^	*p*
Gender (female/male) [*n* (%)]	43 (34.13%)/83 (65.87%)	33 (27.97%)/85 (72.03%)	1.078	0.299
Age (years)	44.63 ± 6.54	45.25 ± 5.77	0.783	0.435
BMI (kg/m²)	23.22 ± 2.25	23.33 ± 2.4	0.359	0.720
Hypertension [*n* (%)]	22 (17.46%)	23 (19.49%)	0.167	0.683
Diabetes [*n* (%)]	10 (7.94%)	7 (5.93%)	0.378	0.539
Smoking history [*n* (%)]	28 (22.22%)	28 (23.73%)	0.078	0.780
Drinking history [*n* (%)]	18 (14.29%)	14 (11.86%)	0.314	0.576
Marital status (married/unmarried or divorced) [*n* (%)]	106 (84.13%)/20 (15.87%)	104 (88.14%)/14 (11.86%)	0.816	0.366
Educational level (high school or below/junior college or above) [*n* (%)]	40 (31.75%)/86 (68.25%)	41 (34.75%)/77 (65.25%)	0.247	0.619

BMI: body mass index; PMMA: polymethylmethacrylate; PMMA-SC: PMMA; sulfate calcium.

The comparison of baseline disease characteristics between the PMMA group and the PMMA-SC group (Table 2) showed no significant differences across all parameters assessed (all *p* > 0.05). Disease courses, post-traumatic vs. postoperative osteomyelitis distribution, mean number of pre-admission operations, bones involved, sinus formation prevalence, and Cierny–Mader type distribution were all similar between the two groups. These findings suggest that the disease characteristics were well-balanced between the two treatment groups, supporting a fair evaluation of treatment efficacy and safety outcomes.

**Table 2.** Comparison of baseline disease characteristics between two groups.

Parameters	PMMA group (*n* = 126)	PMMA-SC group (*n* = 118)	*t* /*χ*^2^/Fisher	*p*
Disease courses (months)	12.78 ± 2.42	12.22 ± 2.18	1.903	0.058
Post-traumatic osteomyelitis/postoperative osteomyelitis [*n* (%)]	73 (57.94%)/53 (42.06%)	71 (60.17%)/47 (39.83%)	0.126	0.723
Mean number of operations done before admission	2.14 ± 1.08	2.19 ± 0.91	0.368	0.713
Bone involved [*n* (%)]			4.882	0.181
Tibia	90 (71.43%)	89 (75.42%)		
Femur	33 (26.19%)	21 (17.80%)		
Humerus	2 (1.59%)	4 (3.39%)		
Radius/ulna	1 (0.79%)	4 (3.39%)		
Sinus formation [*n* (%)]	45 (35.71%)/81 (64.29%)	51 (43.22%)/67 (56.78%)	1.439	0.230
Cierny–Mader type [*n* (%)]			None	0.281
Type 1	27 (21.43%)	23 (19.49%)		
Type 2	0 (0%)	0 (0%)		
Type 3	90 (71.43%)	79 (66.95%)		
Type 4	9 (7.14%)	16 (13.56%)		

PMMA: polymethylmethacrylate; PMMA-SC: PMMA-sulfate calcium.

### 3.2. Postoperative outcomes

The rate of infection control was significantly higher in the PMMA-SC group, with 87.29% of patients achieving infection control compared to 72.22% in the PMMA group (*p* = 0.004) (Table 3). Conversely, the rate of persistent or recurrent infection was higher in the PMMA group at 27.78% versus 12.71% in the PMMA-SC group (*p* = 0.004). Additionally, the reoperation rate was significantly lower in the PMMA-SC group, with 7.63% requiring reoperation compared to 21.43% in the PMMA group (*p* = 0.002). The mean postoperative follow-up duration was marginally longer in the PMMA-SC group (23.75 ± 4.68 months) compared to the PMMA group (22.80 ± 4.52 months), although this difference was not statistically significant (*p* = 0.107). These findings suggest that vancomycin-sulfate calcium may provide superior outcomes in terms of infection control and reduced reoperation rates compared to vancomycin-PMMA in the treatment of chronic osteomyelitis.

**Table 3.** Comparison of postoperative outcomes between two groups.

Parameters	PMMA group (*n* = 126)	PMMA-SC group (*n* = 118)	*t*/*χ*^2^	*p*
Postoperative infection [*n* (%)]				
Infection controlled	91 (72.22%)	103 (87.29%)	8.489	0.004
Persistent or recurrent infection	35 (27.78%)	15 (12.71%)	8.489	0.004
Reoperation rate [*n* (%)]	27 (21.43%)	9 (7.63%)	9.228	0.002
Mean postoperative follow-up (months)	22.80 ± 4.52	23.75 ± 4.68	1.618	0.107

PMMA: polymethylmethacrylate; PMMA-SC: PMMA-sulfate calcium.

It is important to note that the reoperation rates reported in Table 3 refer exclusively to unplanned reoperations performed for persistent infection, recurrent infection, or wound complications. All patients in both groups who received PMMA (with or without calcium sulfate) underwent a planned second-stage surgery for PMMA removal approximately 6–8 weeks after initial implantation; these planned procedures were not counted as reoperations in the analysis of postoperative outcomes.

### 3.3. Efficacy

Unlike Table 3, which reports immediate postoperative infection control and reoperation rates, Table 4 presents a composite efficacy classification (healed/improved/unhealed) based on longer-term clinical, radiological, and laboratory criteria at one year of follow-up. The comparison of treatment efficacy between the PMMA group and the PMMA-SC group (Table 4) revealed significant differences in healing rates, improvement rates, unhealed rates, and total effective rates. The PMMA-SC group demonstrated a significantly higher proportion of healed patients (61.02% vs. 30.95%, *χ*^2^=28.565, *p* < 0.001) and a lower proportion of unhealed patients (7.63% vs. 29.37%, *χ*^2^=28.565, *p* < 0.001). Although the proportion of improved patients was slightly lower in the PMMA-SC group (31.36% vs. 39.68%), the total effective rate (healed + improved) was significantly higher in the PMMA-SC group (92.37% vs. 70.63%, *χ*^2^=18.822, *p* < 0.001). These results indicated that the PMMA-SC intervention was more effective, particularly in achieving healed status and reducing the proportion of unhealed cases, compared to the PMMA group.

**Table 4.** Comparison of efficacy between two groups.

Parameters	PMMA group (*n* = 126)	PMMA-SC group (*n* = 118)	*χ* ^2^	*p*
			28.565	<0.001
Healed [*n* (%)]	39 (30.95%)	72 (61.02%)		
Improved [*n* (%)]	50 (39.68%)	37 (31.36%)		
Unhealed [*n* (%)]	37 (29.37%)	9 (7.63%)		
Total effective [*n* (%)]	89 (70.63%)	109 (92.37%)	18.822	<0.001

PMMA: polymethylmethacrylate; PMMA-SC: PMMA-sulfate calcium.

### 3.4. Inflammatory indicators

The comparison of hematologic cellular indices and systemic inflammatory ratios between the PMMA group and the PMMA-SC group (Table 5) revealed no significant differences at baseline for WBC, neutrophils, lymphocytes, platelets, NLR, PLR, or SII (all *p* > 0.05), indicating similar initial profiles. Two months after treatment, significant differences emerged: the PMMA-SC group showed significantly lower WBC (*t* = 3.060, *p* = 0.002), neutrophil counts (*t* = 3.163, *p* = 0.002), and higher lymphocyte counts (*t* = 3.301, *p* = 0.001), suggesting a more robust recovery of adaptive immune function. Additionally, platelet counts were lower in the PMMA-SC group (*t* = 2.688, *p* = 0.008). Systemic inflammatory markers also showed significant differences, with the PMMA-SC group having a lower NLR (*t* = 4.221, *p* < 0.001), PLR (*t* = 3.289, *p* = 0.001), and SII (*t* = 4.741, *p* < 0.001). These findings indicated that the PMMA-SC intervention was associated with significant improvements in hematologic parameters and reductions in systemic inflammation compared to the PMMA group two months after treatment.

**Table 5.** Comparison of hematologic cellular indices and systemic inflammatory ratios between two groups.

Parameters	PMMA group (*n* = 126)	PMMA-SC group (*n* = 118)	*t*	*p*
WBC (×10⁹/L)				
Baseline	9.85 ± 2.11	10.02 ± 2.34	0.619	0.537
2 months after treatment	7.12 ± 1.45	6.58 ± 1.29	3.060	0.002
Neutrophils (×10⁹/L)				
Baseline	7.21 ± 1.89	7.35 ± 1.71	0.604	0.546
2 months after treatment	4.55 ± 1.12	4.12 ± 0.98	3.163	0.002
Lymphocytes (×10⁹/L)				
Baseline	1.58 ± 0.43	1.62 ± 0.47	0.741	0.460
2 months after treatment	1.91 ± 0.41	2.08 ± 0.39	3.301	0.001
Platelets (×10⁹/L)				
Baseline	292.45 ± 51.67	285.91 ± 52.34	0.982	0.327
2 months after treatment	243.33 ± 40.21	229.76 ± 38.55	2.688	0.008
NLR				
Baseline	4.57 ± 0.75	4.52 ± 0.64	0.544	0.587
2 months after treatment	2.33 ± 0.48	2.06 ± 0.52	4.221	<0.001
PLR				
Baseline	182.36 ± 45.22	176.84 ± 48.71	0.917	0.360
2 months after treatment	126.18 ± 35.67	112.55 ± 28.94	3.289	0.001
SII				
Baseline	1250.23 ± 350.54	1205.82 ± 320.37	1.031	0.304
2 months after treatment	680.53 ± 110.46	615.65 ± 102.81	4.741	<0.001

PMMA: polymethylmethacrylate; PMMA-SC: PMMA-sulfate calcium; WBC: white blood cell count; NLR: neutrophil-to-lymphocyte ratio; PLR: platelet-to-lymphocyte ratio; SII: systemic immune-inflammation index.

Although the absolute differences in some markers (e.g. ESR: 1.53 mm/h; CRP: 0.47 mg/L) were modest, they were accompanied by consistent improvements across multiple interrelated parameters, suggesting a clinically meaningful trend toward reduced systemic inflammation in the PMMA-SC group.

**Figure 1.**
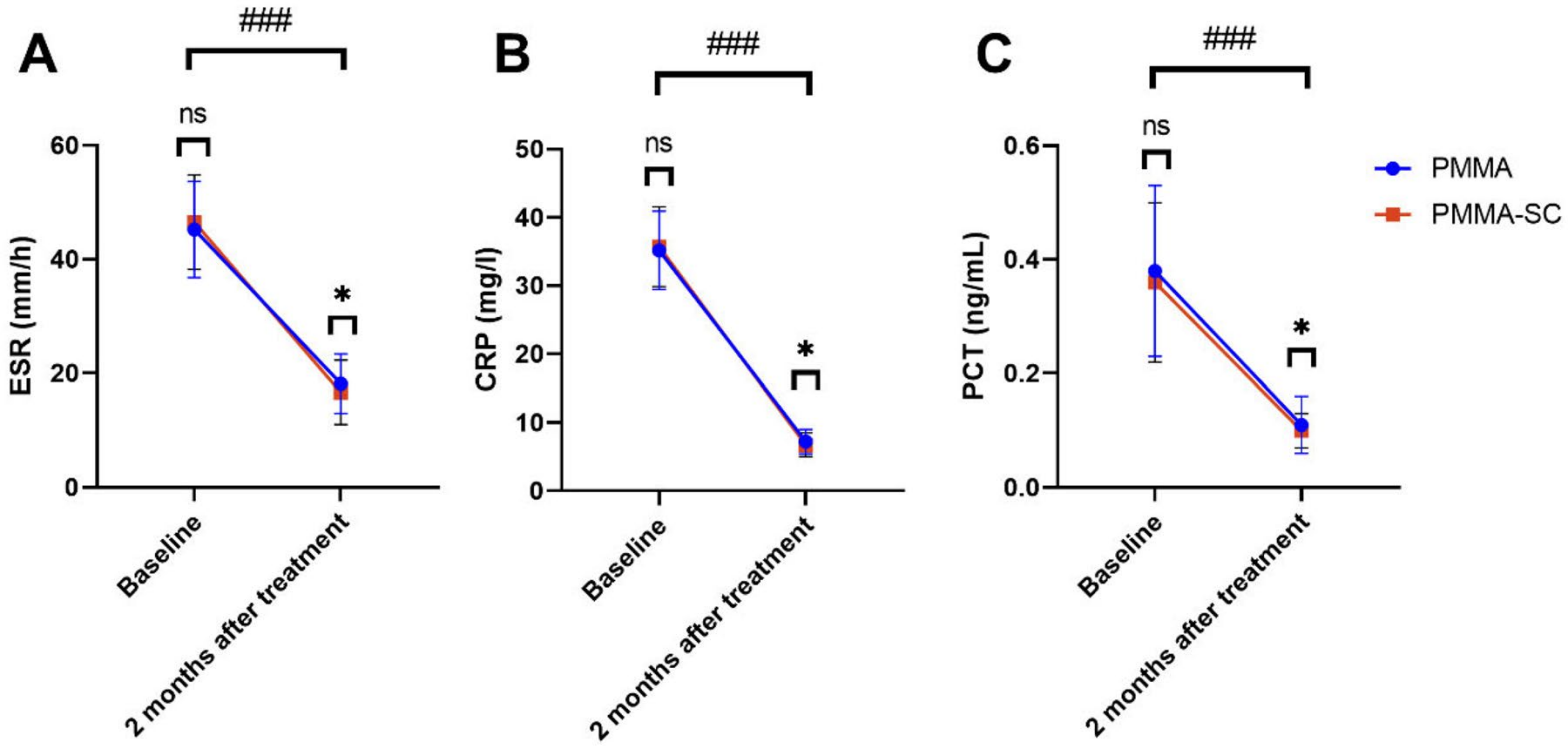
Comparison of serum inflammatory proteins between two groups. (A) ESR; (B) CRP; (C) PCT. ns: no significant difference, * *p* < 0.05 for PMMA vs. PMMA-SC. # *p* < 0.001 for within-group changes from baseline to 2 months. ESR: erythrocyte sedimentation rate; CRP: C-reactive protein; PCT: procalcitonin; PMMA: polymethylmethacrylate; PMMA-SC: PMMA-sulfate calcium.

The comparison of serum inflammatory proteins between the PMMA group and the PMMA-SC group (Figure 1) revealed no significant differences at baseline for ESR, CRP, or PCT (all *p* > 0.05), indicating similar initial inflammatory profiles. Two months after treatment, significant differences were observed: the PMMA-SC group showed significantly lower ESR (16.64 ± 5.64 vs. 18.17 ± 5.18, *t* = 2.213, *p* = 0.028), CRP (6.74 ± 1.72 vs. 7.21 ± 1.76, *t* = 2.116, *p* = 0.035), and PCT (0.10 ± 0.03 vs. 0.11 ± 0.05, *t* = 2.100, *p* = 0.037). These results indicated that the PMMA-SC intervention was associated with significant reductions in serum inflammatory markers compared to the PMMA group two months after treatment.

### 3.5. QoL

**Figure 2.**
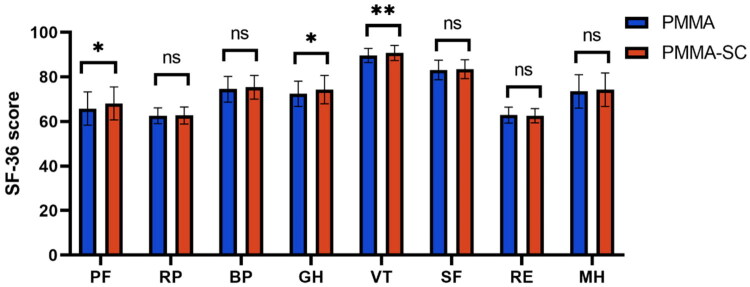
Comparison of SF-36 score between two groups. ns: no significant difference, * *p* < 0.05. SF-36: the Short Form-36 Health Survey Questionnaire; PF: physical functioning; RP: role-physical; BP: bodily pain; GH: general health; VT: vitality; SF: social functioning; RE: role-emotional; MH: mental health; PMMA: polymethylmethacrylate; PMMA-SC: PMMA-sulfate calcium.

The PMMA-SC group demonstrated superior performance in the PF domain, with a mean score of 68.15 ± 7.42 compared to 65.82 ± 7.48 in the PMMA group (*p* = 0.015) (Figure 2). GH scores were also higher in the PMMA-SC group (74.33 ± 6.41) compared to the PMMA group (72.42 ± 5.72, *p* = 0.015). In the VT domain, the PMMA-SC group scored 90.78 ± 3.38, significantly higher than the 89.62 ± 3.13 observed in the PMMA group (*p* = 0.006). Other domains, namely RP, BP, SF, RE, and MH, exhibited no significant differences between the groups, with *p*-values ranging from 0.232 to 0.765. These results suggest that vancomycin-sulfate calcium has a more positive impact on certain QoL aspects, notably PF, GH, and VT, in patients with chronic osteomyelitis when compared to vancomycin-PMMA. While the differences in PF, GH, and VT scores were relatively small (2–3 points), such improvements are considered clinically relevant in chronic disease populations and may reflect meaningful enhancements in daily functioning.

### 3.6. Complications

The incidence of persistent drainage from the wound was notably higher in the PMMA group, affecting 8.73% of patients compared to 0% in the PMMA-SC group (*p* = 0.001) (Table 6). Persistent drainage from the wound was defined as continuous exudate requiring dressing changes for more than 14 days postoperatively, in the absence of hematoma formation or superficial surgical site infection. This complication was observed in 11 patients (8.73%) in the PMMA group but in no patients (0%) in the PMMA-SC group (*p* = 0.001). Transient drainage resolving within 14 days occurred in both groups but was not classified as a complication and was managed conservatively with dressing changes. While nail infections related to external fixation occurred only in the PMMA group with a rate of 3.97%, this difference was not statistically significant (*p* = 0.083). Cases of thrombosis and stiffness showed no significant variance between the groups, with thrombosis observed in 1.59% of PMMA patients and 2.54% of PMMA-SC patients (*p* = 0.941), and stiffness in 9.52% versus 5.08% (*p* = 0.185). Fracture incidence was also similar, occurring in 2.38% of the PMMA group and 0.85% of the PMMA-SC group (*p* = 0.661). Overall, the total complication rate was significantly lower in the PMMA-SC group at 8.47% compared to 26.19% in the PMMA group (*p* < 0.001), indicating a superior safety profile for vancomycin-sulfate calcium in the management of chronic osteomyelitis.

**Table 6.** Comparison of complications between two groups.

Parameters	PMMA group (*n* = 126)	PMMA-SC group (*n* = 118)	*χ* ^2^	*p*
Persistent drainage from the wound [*n* (%)]	11 (8.73%)	0 (0%)	10.788	0.001
Nail infection of external fixation [*n* (%)]	5 (3.97%)	0 (0%)	3.008	0.083
Thrombosis [*n* (%)]	2 (1.59%)	3 (2.54%)	0.005	0.941
Stiffness [*n* (%)]	12 (9.52%)	6 (5.08%)	1.757	0.185
Fracture [*n* (%)]	3 (2.38%)	1 (0.85%)	0.192	0.661
Total [*n* (%)]	33 (26.19%)	10 (8.47%)	13.174	<0.001

PMMA: polymethylmethacrylate; PMMA-SC: PMMA-sulfate calcium.

### 3.7. Multivariate analysis of predictors for treatment effectiveness

The multivariate logistic regression analysis (Table 7) demonstrated that the PMMA-SC treatment approach was a significant protective factor for achieving ‘healed’ and ‘improved’ outcomes, with an odds ratio (OR) of 3.853 (95% CI: 1.982–7.499, *p* < 0.001). Other factors, including age, diabetes, and smoking history, did not show significant associations with treatment effectiveness (all *p* > 0.05). This indicates that the PMMA-SC intervention may increase the likelihood of successful treatment outcomes compared to the PMMA group.

**Table 7.** Multivariate logistic regression analysis of factors associated with treatment effectiveness.

Parameters	Category	OR	95% CI	*p*
Treatment approach	PMMA-SC (vs. PMMA)	3.853	1.982–7.499	<0.001
Age	Per year increase	0.991	0.953–1.035	0.562
Diabetes	Yes (vs. No)	0.825	0.263–1.994	0.527
Smoking history	Yes (vs. No)	1.223	0.653–2.686	0.442

PMMA: polymethylmethacrylate; PMMA-SC: PMMA-sulfate calcium; OR: odds ratio; CI: confidence interval.

## 4. Discussion

Chronic osteomyelitis poses a significant therapeutic challenge due to its complex pathophysiology and the high recurrence rates associated with conventional treatments. This study aimed to evaluate the comparative impact of vancomycin-loaded PMMA versus vancomycin-loaded PMMA and vancomycin sulfate calcium (PMMA-SC) on inflammatory response and clinical outcomes in patients with chronic osteomyelitis. The findings suggest that the addition of sulfate calcium to vancomycin treatment may enhance infection control, reduce reoperation rates, and improve overall QoL. Importantly, these improvements are accompanied by significant changes in systemic inflammation markers, indicating potential mechanisms underlying the observed benefits.

The superior infection control and reduced reoperation rate observed in the PMMA-SC group suggest a more effective eradication of chronic osteomyelitis compared to the traditional vancomycin-PMMA treatment. One possible explanation for these findings lies in the enhanced delivery of vancomycin to the infected site. Calcium sulfate acts not only as a vehicle for antibiotics but also as a biodegradable material that gradually dissolves, releasing antibiotics over a sustained period compared to PMMA, which releases the antibiotic in a more limited, initial burst [21]. This sustained release could maintain therapeutic concentrations of vancomycin in the local microenvironment for an extended period, thereby facilitating more effective suppression of bacterial growth and potentially reducing recurrence rates [22–24].

The significant improvements in hematologic parameters and reductions in systemic inflammation observed in the PMMA-SC group provide valuable insights into the underlying mechanisms of action. Systemic inflammation plays a critical role in the progression of chronic osteomyelitis, as it can lead to prolonged activation of immune cells and subsequent tissue damage [25]. The observed decreases in white blood cell (WBC) counts and neutrophil levels, along with increases in lymphocyte counts, suggest a shift towards a more balanced immune response. This modulation of the immune system may help mitigate excessive inflammation and promote a more favorable environment for tissue repair [26]. A high NLR and SII reflect neutrophilia-driven inflammation coupled with relative lymphopenia, a hallmark of immune dysregulation in chronic infections. The dramatic reduction in these composite indices within the PMMA-SC group indicates a shift from a pro-inflammatory, immune-exhausted state towards immunological homeostasis. This shift was driven by a dual effect: a more substantial decrease in neutrophil counts, signifying the containment of acute inflammatory responses, coupled with a significant recovery in absolute lymphocyte count. The recovery of lymphocytes is a critical indicator of the restoration of adaptive immune competence, which is essential for long-term infection control and tissue repair [27,28].

The improvements in serum inflammatory proteins further support the anti-inflammatory effects of the PMMA-SC intervention. Chronic osteomyelitis is characterized not only by local tissue damage but also by a persistent systemic inflammatory state that can impair overall immune competence. The significantly greater reduction in the classic inflammatory markers ESR and CRP in the PMMA-SC group aligns with this clinical improvement. Moreover, the more pronounced decrease in procalcitonin (PCT) levels, a highly specific marker for systemic bacterial infection, suggests that the PMMA-SC combination may provide a more effective control of the underlying bacterial load, thereby directly mitigating the primary driver of the inflammatory cascade. These changes are likely mediated by the combined actions of sulfate calcium and vancomycin, which together may suppress pro-inflammatory cytokine production and enhance anti-inflammatory pathways. For instance, sulfate calcium has been shown to inhibit nuclear factor kappa-light-chain-enhancer of activated B cells (NF-κB) signaling, a key pathway involved in inflammation. By modulating NF-κB activity, sulfate calcium could reduce the expression of pro-inflammatory genes and promote a more controlled immune response. Similarly, vancomycin’s antibacterial effects help eliminate pathogens, thereby reducing the source of inflammation and allowing for better resolution [27,29]. However, these mechanisms remain speculative in the context of human osteomyelitis and require direct histological and molecular confirmation.

Additionally, the biomechanical properties of calcium sulfate may provide indirect benefits that facilitate healing and infection clearance [30]. The resorbable nature of calcium sulfate means that it was eventually replaced by new bone growth, as assessed by serial plain radiographs during follow-up, potentially restoring anatomical structure and function more naturally than PMMA, which remains in the body indefinitely and can sometimes act as a foreign body conducive to future bacterial colonization [31]. This feature of calcium sulfate could support not only the biochemical eradication of infections but also functional recovery, as evidenced by the higher QoL scores, particularly in domains such as PF and VT.

QoL improvements observed in the PMMA-SC group, particularly in PF, GH, and VT domains, further highlight the clinical benefits of this intervention. Chronic osteomyelitis significantly impacts patients’ daily lives, leading to pain, functional limitations, and psychological distress. By improving infection control and reducing systemic inflammation, the PMMA-SC treatment may alleviate these burdens, resulting in better QoL outcomes. Enhanced physical function and vitality are particularly important for patients recovering from chronic conditions, as they enable a faster return to normal activities and improved overall well-being. These improvements are likely driven by the combined effects of reduced inflammation and better infection control, which together create a more conducive environment for recovery.

In terms of complications, the PMMA-SC group exhibited significantly fewer adverse events, particularly noting an absence of persistent wound drainage and lower incidences of implant-related infections. This finding could be attributed to the nature of the materials used; PMMA does not undergo bioresorption and can potentially act as a nidus for infection if improperly incorporated or colonized by pathogens. Contrastively, calcium sulfate’s resorbable property might reduce sites for bacterial adherence and biofilm formation, thus lowering complication rates [32–34]. Furthermore, the minimal invasiveness of implanting calcium sulfate, combined with its complete bioresorption, minimizes the surgical burden and potential for postsurgical infections requiring reoperations. The complete absence of persistent wound drainage (>14 days) in the PMMA-SC group, while notable, should be interpreted with caution given the retrospective design and relatively modest sample size. However, this finding is consistent with the biodegradable nature of calcium sulfate, which may reduce local tissue irritation compared to permanent PMMA.

An important consideration with biodegradable antibiotic carriers such as calcium sulfate is the potential risk of prolonged exposure to sub-minimal inhibitory concentration (sub-MIC) antibiotic levels during the late elution phase. While the sustained release profile of vancomycin from calcium sulfate provides extended therapeutic coverage, the declining concentrations over time may fall below the MIC for certain pathogens before complete bacterial eradication is achieved. Sub-MIC antibiotic exposure has been associated with increased biofilm formation and the potential selection of resistant bacterial subpopulations in experimental studies. In our series, the significantly lower recurrence rate in the PMMA-SC group suggests that any theoretical risk of sub-MIC exposure did not translate into clinically evident treatment failure. However, this remains an important consideration, and future studies should incorporate serial local antibiotic concentration measurements to characterize the complete elution profile and confirm that concentrations remain above MIC for the duration of treatment. Additionally, extended microbiological surveillance for emerging resistance would be valuable in longer-term follow-up studies.

Nonetheless, the study’s retrospective nature presents limitations. The treatment allocation in this retrospective study was not randomized and may have been influenced by surgeon preference and the chronological period of treatment, with PMMA-SC increasingly adopted after 2021. This temporal introduction and reliance on surgeon discretion make reliable differentiation between the two groups challenging. Non-randomized allocation introduces potential selection bias. The observed demographic and comorbidity comparability between groups is not surprising, as both cohorts were drawn from the same institution over consecutive time periods; such comparability reflects the stability of the patient population rather than an absence of allocation bias. Consequently, Tables 1 and 2 do not represent two inherently distinct patient populations but rather sequential cohorts from the same clinical setting, which precludes claims of true ‘matching’ and underscores the historical comparison design. Additionally, defect size and morphology may have influenced the choice of PMMA-SC, as calcium sulfate offers superior osteoconductive properties for larger bone defects. These selection bias cannot be fully excluded despite the well-balanced baseline characteristics between groups. Although measures were taken to ensure demographic and baseline characteristic parity, inherent variability and confounding factors cannot be entirely ruled out. The absence of microbiological stratification and pathogen-specific subgroup analysis limits our ability to assess efficacy against different pathogens. The sample size, although relatively large, precluded extensive multivariable adjustments and increased the risk of type I error due to multiple comparisons. Future prospective studies, possibly including randomized controlled trials, were necessary to corroborate these findings and further elucidate the long-term benefits and mechanistic underpinnings of vancomycin sulfate calcium in osteomyelitis treatment. Furthermore, while our hematological indices provide valuable insights into systemic inflammatory and immune status, they do not elucidate the precise local cellular and molecular mechanisms at the bone infection site. The lack of local tissue-level inflammatory data restricts our understanding of the precise mechanisms at the infection site. Only two time points were assessed for inflammatory markers, and systemic markers may not fully reflect local bone inflammation. The follow-up duration, while adequate for assessing recurrence, may not capture very late complications or relapses. Although our study did not include a formal correlation analysis between biochemical response and patient-reported outcomes – as the primary objective was to compare treatment groups rather than to identify predictive biomarkers – the observed improvements in both inflammatory markers and QoL scores in the PMMA-SC group suggest potential interrelationships. Future prospective studies with pre-specified correlational analyses could help determine which inflammatory parameters most strongly predict successful clinical and functional outcomes, potentially enabling early identification of patients at risk for treatment failure.

We fully acknowledge that the methodological challenges of this historical comparison – including non-randomized allocation, temporal bias, and surgeon preference – may have introduced confounding that could inflate the observed differences in clinical outcomes, quality of life, and complications. Despite the statistically significant findings, the possibility of type I error cannot be excluded. As such, our conclusions should be interpreted as hypothesis-generating rather than definitive. The observed superiority of PMMA-SC over PMMA alone requires confirmation in well-designed prospective randomized controlled trials before clinical adoption can be recommended.

## 5. Conclusion

In conclusion, this study demonstrates that the combination of vancomycin-loaded calcium sulfate with PMMA may offer superior clinical efficacy over vancomycin-PMMA alone in the management of chronic osteomyelitis. The enhanced infection control and reduced complication profile were underpinned by a more favorable trajectory of inflammatory response, as evidenced by a significant attenuation of both serological and cellular inflammatory markers. Critically, the observed modulation of systemic immune-inflammation indices suggests a potential role for the biodegradable composite in promoting a shift from a pro-inflammatory state towards immune homeostasis, though these findings are hypothesis-generating. Prospective randomized trials are warranted to confirm these observations and establish the efficacy and safety of the PMMA-SC strategy before widespread clinical adoption.

## Data Availability

The datasets used during the present study are available from the corresponding author upon reasonable request.
